# Making wider use of the world's most widely used vaccine: Bacille Calmette–Guérin revaccination reconsidered

**DOI:** 10.1098/rsif.2013.0365

**Published:** 2013-10-06

**Authors:** Christopher Dye

**Affiliations:** World Health Organization, Avenue Appia, CH1211 Geneva 27, Switzerland

**Keywords:** tuberculosis, immunization, Bacille Calmette–Guérin, cost-effectiveness, revaccination, adolescents

## Abstract

Approximately 100 million newborn children receive Bacille Calmette–Guérin (BCG) annually, because vaccination is consistently protective against childhood tuberculous meningitis and miliary TB. By contrast, BCG efficacy against pulmonary TB in children and adults is highly variable, ranging from 0% to 80%, though it tends to be higher in individuals who have no detectable prior exposure to mycobacterial infections, as judged by the absence of delayed-type hypersensitivity response (a negative tuberculin skin test, TST). The duration of protection against pulmonary TB is also variable, but lasts about 10 years on average. These observations raise the possibility that BCG revaccination, following primary vaccination in infancy, could be efficacious among TST-negative adolescents as they move into adulthood, the period of highest risk for pulmonary disease. To inform continuing debate about revaccination, this paper assesses the effectiveness and cost-effectiveness of revaccinating adolescents in a setting with intense transmission—Cape Town, South Africa. For a cost of revaccination in the range US$1–10 per person, and vaccine efficacy between 10% and 80% with protection for 10 years, the incremental cost per year of healthy life recovered (disability-adjusted life years, DALY) in the vaccinated population lies between US$116 and US$9237. The intervention is about twice as cost-effective when allowing for the extra benefits of preventing transmission, with costs per DALY recovered in the range US$52–$4540. At 80% efficacy, revaccination averted 17% of cases. Under the scenarios investigated, BCG revaccination is cost-effective against international benchmarks, though not highly effective. Cost-effectiveness ratios would be more favourable if we also allow for TB cases averted by preventing transmission to HIV-positive people, for the protection of HIV-negative people who later acquire HIV infection, for the possible non-specific benefits of BCG, for the fact that some adolescents would receive BCG for the first time, and for cost sharing when BCG is integrated into an adolescent immunization programme. These findings suggest, subject to further evaluation, that BCG revaccination could be cost-effective in some settings.

## 1. Introduction

In the aftermath of disappointing results from a phase IIb trial of candidate TB vaccine MVA85A [1], and while awaiting the results of further TB vaccine trials, this paper considers whether the vaccine we already have, Bacille Calmette–Guérin (BCG), is being used to its full effectiveness. Approximately 100 million newborn children are vaccinated with BCG every year, but vaccination and revaccination of older children and adults has largely been discontinued because it is thought not to be sufficiently effective or cost-effective [2–4]. The aim of this study is to reconsider whether it would be effective and cost-effective, under some circumstances, to offer BCG revaccination to adolescents, as a booster to primary vaccination in infancy. If BCG revaccination is, indeed, cost-effective, it might be provided as one element of an immunization programme to protect adolescents against a variety of common, vaccine-preventable infectious diseases, and as a platform for the introduction of future TB vaccines. This analysis does not, on its own, suggest any changes to BCG revaccination policy, and is not a policy statement of the World Health Organization. Rather, it suggests that the costs and effectiveness of BCG revaccination are worth further consideration in some settings, preferably backed by further evidence from clinical trials.

BCG has consistently high efficacy against TB meningitis and miliary disease in children under 5 years of age, and neonatal vaccination is cost-effective in high-burden countries for this reason alone [5]. By contrast, the protective efficacy of BCG against pulmonary TB is variable, ranging from zero (or even negative) to about 80% [6,7]. Efficacy tends to be higher among individuals who respond negatively or weakly to a tuberculin skin test (TST, inoculation of a multi-antigenic purified mycobacterial protein derivative that stimulates a delayed-type hypersensitivity response) [7], suggesting that they have not previously been exposed to infection with *Mycobacterium tuberculosis* or other non-tuberculous mycobacteria (NTMs). For example, after 15 years of follow-up to a randomized controlled trial of BCG vaccination in South India, protection averaged 32% (95% CI: 3–52%), among people who were initially non-responsive to NTMs on a TST [8]. There was no significant protection in people previously exposed to NTMs. It is generally assumed that vaccination does not protect people who are TST-positive, whether owing to natural mycobacterial infection or to primary BCG vaccination [4].

Where BCG is protective, the duration of protection is variable. The average is about 10 years [9], but approximately 50% protection has been observed 15–20 years after vaccination in Brazil [10], and 40–50 years after vaccination in American Indians and Alaskan natives [11,12].

There are fewer data on the efficacy of BCG revaccination. Both primary vaccination and revaccination protected children and adults against leprosy in Karonga District, Malawi, but neither the first vaccination nor repeat vaccination was protective against TB [13]. Similarly, revaccination did not protect children 6–9 years old from TB in Hong Kong [14]. In Brazil, revaccination of children 7–14 years old gave 19% (95% CL: 3–33%) protection on average in coastal Salvador over 9 years of follow-up, but there was no evidence of protection in equatorial Manaus [15]. These results are consistent with the hypothesis that BCG vaccination has higher efficacy in more temperate areas with lower exposure to NTMs. There is apparently only one published report of BCG revaccination in people with negative or small TST responses: a retrospective analysis carried out in Poland from 1965 to 1977 found a ‘higher incidence’ (details unknown) over the 12-year period among individuals who were not revaccinated [2].

Under some circumstances, BCG vaccination prevents mycobacterial infection (probably *M. tuberculosis*) in addition to active disease [16–18], and so does revaccination [19]. Thus, in a prison population in Taiwan, there were fewer infections (measured as positive responses on a QuantiFERON TB gold test) among inmates who had greater than or equal to two BCG scars, when compared with those who had one scar or no scar [19]. BCG also confers non-specific benefits beyond mycobacterial diseases (leprosy and Buruli ulcer), ameliorating atopic disorders, intestinal nematode infections, bladder cancer and malignant melanoma, and augmenting immune responses to other vaccines [20,21].

The maximum efficacy of BCG vaccination is comparable with the immune protection enjoyed by individuals who carry long-standing (latent) mycobacterial infections. Individuals with a durable positive TST (more than 1–2 years) are those who did not progress to active TB soon after infection. Their apparent immunity could be a pre-infection characteristic of either host or pathogen, or a host response to infection. From the perspective of developing new vaccines, the distinction is important: the latter implies that infection stimulates a protective immune response, which might be replicated with a new vaccine; the former does not. Whichever explanation is correct, a meta-analysis of a series of studies shows that the risk of TB following infection in TST-positive individuals averages only 21% (95% CI : 14–30%) of the risk in TST-negative individuals [22]. This is consistent with some estimates obtained from mathematical modelling [23–25] but lower than others [26,27].

It has been pointed out that a negative TST (no detectable induration, or an induration below a defined threshold size) in any individual does not guarantee any of the following: (i) no previous exposure to *M. tuberculosis* infection or NTMs, (ii) susceptibility to infection and disease or (iii) that BCG would be protective against infection or disease [28,29]. Nevertheless, a negative TST is associated with the potential for protection by BCG, and a positive TST is associated with protection from reinfection. These are correlates of immunity, albeit imperfect correlates.

Thus, in some settings, the absence of a signal from NTMs, together with the possibility that protection from neonatal vaccination lasts only 10 years, raises the question of whether it would be cost-effective to revaccinate TST-negative adolescents as they move into adulthood, the period of highest risk for pulmonary TB. This paper addresses that question by evaluating the costs in relation to the potential benefits, taking as an example one setting with intense TB transmission, namely Cape Town, South Africa.

## 2. Data and methods

### 2.1. Tuberculosis infection and disease in Cape Town

**Figure 1.**
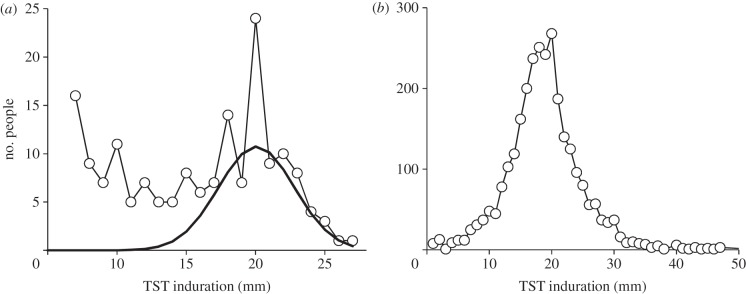
Distributions of tuberculin skin-test responses in children in (*a*) North India [30] and (*b*) Cape Town, South Africa [31]. The solid line in (*a*) is an estimate of the number of children infected with *M. tuberculosis*, as distinct from those showing cross reactions with non-tuberculous mycobacteria or primary BCG vaccination at smaller induration sizes.

TST surveys carried out in tropical and equatorial latitudes usually indicate that children are exposed both to *M. tuberculosis* and NTMs: the delayed-type hypersensitivity response typically shows a dominant signal attributable to *M. tuberculosis* infection around an induration size of 20 mm, and a spread of weaker responses thought to be due to primary BCG vaccination and exposure to NTMs (figure 1*a*). At latitudes away from the equator, such as Cape Town, the weaker background signal from neonatal BCG and NTM exposure is almost non-existent (figure 1*b*).

**Figure 2.**
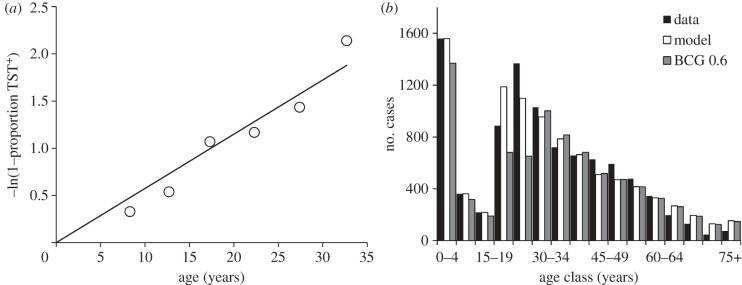
(*a*) The proportion of people (*p*) with a positive TST by age, linearized with the transformation –ln(1−*p*). (*b*) The number of HIV-negative TB cases reported from Cape Town in 2009 (black), the number estimated by fitting model (2.1)–(2.5) (white), and the number obtained when BCG revaccination has efficacy 0.6 (grey). Adapted from Wood *et al.* [32,33].

Based on the proportion of TST responses that are positive in children of different ages, Wood *et al.* [32] have measured the annual risk of infection in Cape Town (figure 2*a*). They have also reported the numbers of TB cases by age and HIV status (figure 2*b*) [33]. The total number of 9290 new cases observed in a cohort of approximately 61 890 HIV-negative people of all ages (309 448 divided by 5 for the age class 0–4 years) represents a lifetime risk of developing TB of 14%, with the reported incidence rate of new cases peaking at 0.45% per year in the age group 20–24 years. The true incidence of TB in the HIV-negative population is probably higher than this [34], but the analysis in this paper is carried out with the reported data. The incidence of new TB cases among HIV-positive people in Cape Town is much higher than among HIV-negatives, reported to be 4–5% annually in all age groups [33].

The estimated coverage of neonatal BCG vaccination was 78% for South Africa nationally in 2011, but varied between 60% and 89% annually between 2000 and 2011 [35]. Most children would therefore have benefited from whatever protection BCG offers against TB meningitis, miliary disease and pulmonary TB. However, a proportion of children enter adolescence and adulthood without being vaccinated.

The empirical review in §1 indicates that the efficacy of BCG revaccination is unpredictable. And, because BCG is nowadays not typically used in adolescents, the cost of revaccination is also unknown. Nevertheless, we can explore the potential cost-effectiveness of revaccination by placing reasonable bounds on costs and efficacy, as follows.

### 2.2. Modelling the potential effects of Bacille Calmette–Guérin revaccination

This analysis focuses on the population of HIV-negative adolescents and adults greater than or equal to 15 years old: this population is assumed to be responsible for all transmitted infections, and teenagers are the target for revaccination. The data in figure 2*b* were collected across all ages classes in Cape Town in 1 year (2009). If we assume that the number of people of different ages and the *M. tuberculosis* infection rates by age are stable (as did Wood *et al.*), then figure 2*b* depicts the fate of a cohort of individuals, with respect to developing active tuberculosis, through the course of their lives.

For this analysis, the fate of a cohort of individuals from 15 years of age onwards is described by the following ordinary differential equations:2.1
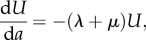
2.2
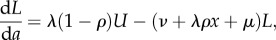
2.3
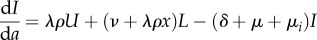
2.4
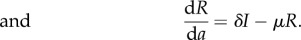
Infection is acquired by uninfected individuals (*U*) at rate *λ* per person per year; this is called the force of infection or the annual risk of infection. Infected individuals either progress rapidly (proportion *ρ*) to active TB (*I*) or (proportion 1 − *ρ*) move into the latent state (*L*), from where they may later ‘reactivate’ at *per capita* rate *ν*. Individuals carrying a latent infection can also develop TB following reinfection, at rate *λρx*, where *x* is the proportion of latently infected people who are susceptible to reinfection (so proportion 1 − *x* is protected).

The annual risk of infection is generated by active TB cases, *λ* = *βΣC*(*a*)/*N*, where *β* is the average number of infectious contacts made by each new case of TB in each age group, *C*(*a*), and *ΣC*(*a*) is the sum of incident cases across all age groups in the population of size *N*. Then, *β* can be estimated from the data as the ratio *λN*/*ΣC*(*a*).

The slope of the regression line in figure 2*a* gives an estimated annual risk of infection of 5.74% per year (*λ* = 0.0574, 95% CL: 0.003). There is evidence that the risk of infection increases with age [36], but it is taken to be constant for the purposes of this analysis. In Cape Town in 2009, the annual risk of infection *λ* = 0.0574 per year was generated by 7152 reported cases among people greater than or equal to 15 years old in a population of 2.3 million people. Each new case was therefore responsible for *β* = 27.6 infections during the infectious period. In fact, there were probably more new cases than reported, in addition to recurrent cases, so this gives an overestimate of *β*. However, this value of *β* is an internally consistent estimate that allows the model to fit the data.

Individuals are lost from the cohort through mortality due to factors other than TB at *per capita* rate *μ* per year. The distribution of people greater than or equal to 15 years by age in Cape Town, if stable, implies that the annual mortality rate averages 0.032 per year (95% CL: 0.005), or a life expectancy of 31 years. Individuals with active TB suffer an additional mortality rate *μ*_*i*_ per year, which is set at 0.2 per year (95% CL 0.05) based on previous studies [23]. In this model, TB cases are saved from death, owing to TB or other causes, by detection and cure at *per capita* rate *δ* per year (set at 0.15). Summing equations (2.1)–(2.4) gives the rate of change of the number of people, *N*, with age *a*:2.5
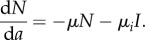


In the steady state, the solution to equation (2.5) is a survival curve for the HIV-negative population of Cape Town, describing the fate of a cohort of individuals from birth to death.

**Figure 3.**
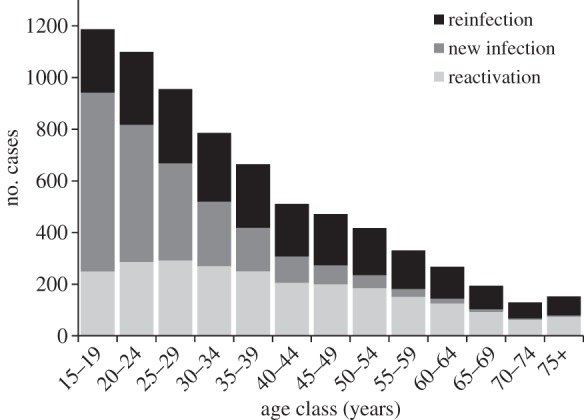
The estimated numbers of new TB cases that arise from new infections, from reinfection and by reactivation in adolescents and adults, Cape Town, South Africa. The numbers are a breakdown of the white bars in figure 2*b*.

Equations (2.1)–(2.5) can be solved numerically and fitted by maximum-likelihood methods to the data in figure 2*b* [37]. TB is a rare event in a large population, so the observed number of cases in each age class is assumed to follow Poisson statistics. Given *k* observed cases and an expected mean of *n*, then likelihood (*k*,*n*) = e^−*n*^*n^k^*/*k*!, where *k*! ≈ *Γ*(*k* + 1). Log(likelihood) is computed for each age class, summed across age classes and the sum maximized by adjusting parameters *ρ* and *ν* with the Solver function in Microsoft Excel. With the point estimates of *δ*, *λ*, *μ* and *μ*_*i*_, given above, the fit provides values of the two free parameters, *ρ* = 0.108 (95% CL: 0.004) and *ν* = 133 per 100 000 per year (95% CL: 10; table 1). The fit generates 7162 cases (95% CL: 880) of TB in HIV-negative people greater than or equal to 15 years, compared with the 7152 observed. The estimated numbers of cases arising from new infections, from reinfection and from reactivation are shown in figure 3. As expected, new cases arise mainly from new infections in adolescents and young adults and mainly by reactivation and reinfection in older adults.

**Table 1.** Parameters used in model (2.1)–(2.5) with their estimated values.

parameter	symbol	value (95% CL)	source
annual risk of infection	*λ*	0.0574 (0.0033) per person per year	estimated in this study
proportion of infections progressing rapidly to active TB	*ρ*	0.108 (0.004) proportion	estimated in this study
rate of reactivation of latent infections	*ν*	133 (9.5) per 100 000 persons per year	estimated in this study
proportion of reinfections that leads to active TB	*x*	0.21 (0.08) proportion	published estimate [22]
mortality rate due to factors other than TB	*μ*	0.032 (0.005) per person per year	estimated in this study
TB mortality rate	*μ*_*i*_	0.2 (0.05) per TB case per year	published estimate [23]

### 2.3. Cost-effectiveness of Bacille Calmette–Guérin revaccination

Having fitted the model to the data, it can be used to explore the potential benefits of BCG revaccination in adolescents, in terms of cases averted and disability-adjusted life years (DALYs) recovered. We assume that all uninfected adolescents (*U*) are revaccinated at age 15, that the proportion protected (vaccine efficacy) ranges from 10% to 80%, in steps of 10% (protected children are transferred from state *U* to state *R*), and that protection lasts for exactly 10 years, after which vaccinees return to their prior uninfected state (*U*). A population coverage of less than 100% (i.e. not all 15 year-olds) would be the same as a lower vaccine efficacy. It is assumed that infected children would not benefit from BCG, and so they are not revaccinated. For each individual vaccinated, the protective efficacy is either zero or 100%. That is, the range of efficacies is captured at the population level, rather than at the individual level, but repeating the analysis at the individual level yields essentially the same results.

To calculate the effect of cutting transmission to children less than 15 years old, all cases arising in that age group are assumed to come from recent infections rather than from the reactivation of old infections. This is a reasonable approximation, because most cases are in children less than 5 years old (figure 2*b*).

In order to compare cost-effectiveness against international benchmarks, the number of cases averted is also converted into DALYs. The number of DALYs lost by each TB case is the sum of years lost through illness (weighted by disability factor 0.5) plus the number of years lost by each case that dies (15 years of healthy life lost, with a case fatality of 5%) [38].

The cost of neonatal BCG vaccination is ≈US$1 per infant [38], but it could be more for adolescents, depending on the mode of delivery. Here, we explore costs in the range US$1–10. A ‘highly cost-effective’ intervention is one that costs fewer US$ per DALY recovered than annual GDP *per capita*, which for South Africa was about US$8000 in 2011 [39].

This analysis of putative cohorts evaluates the difference, owing to BCG revaccination, in numbers of cases arising and DALYs lost in the present steady state compared with a future steady state; it does not track the dynamical change between equilibria. Comparing the two steady-state cohorts gives the number of cases averted and the number of DALYs recovered. Because there is no revaccination programme in place in South Africa, all costs are incremental. The analysis is carried out from the perspective of the government as the provider of health services.

## 3. Results

**Figure 4.**
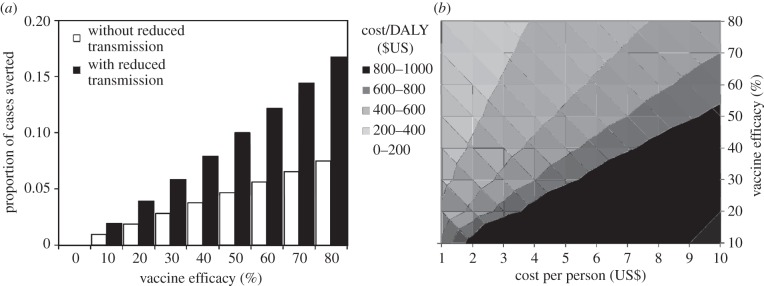
The impact of revaccination measured in terms of (*a*) the proportion of cases averted in the cohort, calculated with (black) and without (white) the effect of reduced transmission, and (*b*) the cost per DALY regained (contours) in terms of the cost of revaccination per person (US$, horizontal axis) and vaccine efficacy (vertical axis), and allowing for the effects of reduced transmission. The darkest area also includes values in excess of US$1000 per DALY regained.

The percentage of cases averted across all age groups (from birth onwards) increases to a maximum of 17% (1554 of 9290 cases) with revaccination efficacy set at 80% (the upper limit in this analysis), when allowing for the direct benefits to vaccinees (7% or 695 cases) plus the consequent reduction in transmitted infections (compare white and black bars, figure 4*a*). Given the uncertainty in parameter estimates in table 1, the 95% CL is 19% of the estimated number of cases averted. The estimate of numbers of cases averted is most sensitive to uncertainty in the value of parameter *x* (which explains 88% of the variation in cases averted), moderately sensitive to *λ*, *ρ* and *ν* (30–70% of the variation) and relatively insensitive to *μ*, *μ*_*i*_ and *σ* (less than 3% of the variation).

Adding in the benefits of reducing transmission roughly doubles the proportion of cases averted—more precisely, by a factor of 2.1 on average across the range of efficacies 10–80%. For a revaccination efficacy of 80%, the annual risk of infection is reduced from 5.7% per year to 4.8% per year, a reduction of almost 1% per year.

The cost per DALY recovered, based on cases saved among vaccinees only (excluding the effect of cutting transmission) ranges from US$116 to US$9237. These estimates can be viewed as the short-term cost-effectiveness of the intervention, given *per capita* revaccination costs in the range US$1–10 and vaccine efficacy between 10% and 80%. Allowing for cases averted through reduced transmission to others in the HIV-negative cohort, the cost per DALY recovered is about half the above (actually by the factor 1/2.1 above), ranging from US$52 to US$4540 (figure 4*b*). To give some specific examples within these ranges, if the efficacy of revaccination is 50%, the cost per DALY recovered is less than US$500 for any revaccination cost less than or equal to US$5 per person, and falls to US$86 if the cost is only US$1 per person. Even with efficacy as low as 30% and a cost of $2 per person, the cost per DALY is around $300. These estimates can be interpreted as the long-term cost-effectiveness of the intervention, which apply once the cohort has reached a new steady state. In the transition between current and future steady states, cost-effectiveness lies between the two sets of estimates given here.

## 4. Discussion

BCG is widely used as a neonatal vaccine because it is low-cost and has consistently high efficacy against severe forms of TB [5]. BCG revaccination has not been recommended for children, adolescents and adults because it appears to confer limited protection against pulmonary TB, and only in some settings [2]. However, the analysis in this paper suggests that BCG revaccination deserves further investigation, at least in locations where there is little exposure to non-tuberculous mycobacteria (as indicated by a TST), and where adolescent children are at a high risk of TB as they move into adulthood.

Considering only the cohort of HIV-negative individuals in Cape Town, a maximum of 17% of cases would be averted. Although the absolute effectiveness of BCG revaccination is thus limited, the intervention is ‘highly cost-effective’ at all the combinations of cost (US$1–10) and efficacy (10–80%) investigated here. For comparison, the estimate of US$52–$4540 per DALY regained by BCG revaccination can be set against a cost per DALY typically less than US$100 for the treatment of active TB [38,40]. If we add in the benefits of preventing TB infections in HIV-positive people, of protecting HIV-negative people who later acquire HIV infection, the possible non-specific benefits of BCG, the fact that some adolescents would be receiving BCG for the first time (having missed out in infancy), and the cost-sharing made possible by including BCG in an adolescent vaccination programme (e.g. with human papilloma virus, measles, mumps and rubella MMR, and diphtheria, tetanus and pertussis DTP vaccines), the balance between costs and effects would be even more favourable [41,42]. To the extent that BCG is efficacious, it also prevents drug-resistant TB, which is costly to manage and associated with higher treatment failure and death rates. None of these extra benefits have been included in the above calculations. Looking to the future, the creation of an adolescent immunization programme would also serve as a platform for the introduction of new vaccines for TB and other infections, as and when they become available. Vaccine MVA85A failed to protect infants from TB [1]; it might have been more successful in adolescents with waning protection 15 years after receiving BCG in infancy [43]. Of course the ratio of costs to effects is just one criterion for judging whether to implement a health intervention; the absolute cost of a programme determines whether it is affordable, and the absolute effectiveness specifies how much of the problem will be solved.

The simple approach to investigating BCG revaccination in this paper inevitably comes with caveats. Apart from seeking better estimates of model parameters (especially *x*, *λ*, *ρ* and *ν*), the assumption that only HIV-negative TB cases transmit infection certainly needs closer scrutiny. At the level of the whole South African population, the huge increase in HIV-positive cases (by an estimated factor of 54.3 between 1990 and 2010) appears to have caused comparatively little increase in the number of HIV-negative cases (by a factor of 1.3 between 1990 and 2010) [44], suggesting that there has been limited transmission from the former to the latter. However, sputum smear-positive disease is frequently found among HIV-positive TB cases in Cape Town [45] and, if these cases are contributing significantly to transmission, the effects of BCG revaccination presented above would be too large. It is possible that additional data will be needed to assess the magnitude of any bias in the calculated effect of revaccination.

The next step would be to assess the efficacy of BCG revaccination given to adolescent children in Cape Town, or in other settings where the balance of costs and effects will probably differ. To minimize the cost of a revaccination trial, and maximize the benefits of adolescent vaccines, a pragmatic design is required, probably including children in a range of age groups. One option is to compare TB incidence in teenagers who test negative for infection (by TST, and perhaps also on an interferon-γ release assay such as QuantiFERON TB gold), with and without BCG, while giving a combination of vaccines other than BCG to all participants. This would miss an opportunity to assess any additional protection given TST-positive individuals (whether due to natural mycobacterial infection or prior BCG vaccination), but present evidence indicates that there is little or no additional protection to be gained in this group. The study might focus on efficacy in individuals only (excluding transmission effects) and rely on routine case reports (cheaper than individual follow-up) to assess the effect on incidence. Another option would be to use the incidence of infection rather than the incidence of disease as an endpoint, because there are many more infections than cases, and there is some evidence that BCG can prevent infection [16–19]. Such a trial is already being planned in Cape Town (A. Ginsberg & W. Hanekom 2013, personal communication).

Because BCG is a live attenuated vaccine, various safeguards are required in clinical trials and in routine practice. HIV-positive people should not be vaccinated [4,46]. Clinical vigilance is required to avoid harm to individuals with subclinical active TB, though these will typically be TST-positive. In anv event, screening to exclude active TB during trials or routine practice would add to costs. Finally, BCG is usually administered intradermally, so clinical staff may require additional training in an adolescent vaccination programme, though percutaneous inoculation may also be acceptable [47].

In sum, there is currently no panacea for TB control, and there is unlikely to be one in the next one to two decades. We must therefore make best use of the mix of interventions available at any one time, devising ways optimally to combine drug treatment and vaccination. The present analysis, on its own, is not intended to change policy; rather it is to suggest that, in some settings and subject to further evaluation, BCG revaccination could be in the mix, along with established (or establishing) programmes of adolescent immunization and TB case detection and drug treatment. To explore the options for wider use of BCG is surely worth the effort—the potential payoff is that we find better ways to protect the health of adolescents as they enter a highly vulnerable period in their lives.
